# Dynamic redistribution of paxillin in bovine osteoblasts stimulated with adenosine 5′-triphosphate

**DOI:** 10.1007/s10735-012-9419-x

**Published:** 2012-05-05

**Authors:** Ann-Sophie Silber, Bastian Pfau, Toh Weng Tan, Ralf Jacob, David Jones, Thomas Meyer

**Affiliations:** 1 Institut für Experimentelle Orthopädie und Biomechanik, Philipps-Universität Marburg, Marburg, Germany; 2 Institut für Optik und Atomare Physik, Technische Universität Berlin, Berlin, Germany; 3 Institut für Zytobiologie und Zytopathologie, Philipps-Universität Marburg, Marburg, Germany; 4 Klinik für Psychosomatische Medizin und Psychotherapie, Georg-August-Universität Göttingen, Von-Siebold-Str. 5, 37075 Göttingen, Germany

**Keywords:** Paxillin, Focal adhesions, Bone remodelling, ATP, Osteoblast

## Abstract

Exposure to extracellular 5′-adenosine triphosphate (ATP) is known to induce membrane blebbing. In this study, we investigated the subcellular distribution of the cytoskeletal adaptor protein paxillin in primary bovine osteoblasts upon stimulation with ATP. Cells expressing a fusion protein of green fluorescent protein (GFP) and paxillin were followed by time-lapse video-microscopy after stimulation with 100 μM ATP. Within 100 s, GFP-paxillin became incorporated in numerous de novo formed focal aggregates localized at the cell periphery. The assembly of individual paxillin-containing aggregates occurred with a mean half-life time of <60 s, whereas their disassembly lasted twice as long. Despite the ongoing presence of ATP, the formation of paxillin aggregates was self-limiting within 25 min. Paxillin clustering was preceded by a transient rise in cytoplasmic calcium transients, which peaked already 20 s after adding ATP. The high mobility of paxillin was confirmed by measuring the dissociation rate of GFP-paxillin at mature focal adhesions, demonstrating the presence of a highly mobile fraction with a mean recovery half-life of 8.2 ± 1.2 s, followed by a slower phase (53 ± 20 s). Thus, both the exchange of paxillin at mature focal adhesions and the increase in intracellular calcium concentrations upon ATP stimulation are very rapid processes, which override the time course of ATP-induced paxillin membrane clustering by one to two orders of magnitude. Our data demonstrate that the transient recruitment of paxillin in membrane protuberances is based on the high intracytoplasmic mobility of unbound paxillin molecules and their rapid focal accumulation.

## Introduction

Over the past years numerous extracellular signalling molecules have been identified to play pivotal roles in the regulation of bone turnover, among them are secreted nucleotides. A growing body of evidence suggested that purinergic stimulation increases proliferation of osteoblasts, suppresses bone mineralization and enhances the bone-resorbing activity of osteoclast (Nakamura et al. 2000; Bowler et al. 2001; Buckley et al. 2002; Orriss et al. 2006, 2007). Extracellular 5′-adenosine triphosphate (ATP) and other nucleotides signal through P2 receptors, formerly termed purinoceptors, which comprise a diverse group of receptors subdivided into P2Y G-protein coupled receptors and P2X ligand-gated ion channels (Bowler et al. 2001; Gallagher and Buckley 2002; Li et al. 2005; Orriss et al. 2007, 2010; Panupinthu et al. 2007; Kaunitz and Yamaguchi 2008). P2Y receptors, consisting of seven hydrophobic membrane-spanning domains separated by alternating extra- and intracellular hydrophilic loops, are widely expressed in both osteoblasts and osteoclasts (Orriss et al. 2006). Activation of P2 receptors is triggered by ATP or other nucleotides, which are released at sites of tissue trauma, injury or fracture by cell lysis. In rat osteoblasts, hypoxia has been shown to affect purinergic signalling by increasing vesicular ATP release (Lew and White 1987; Leung et al. 1989; Orriss et al. 2009). However, nucleotides may also be released constitutively by osteoblasts in a non-lytic manner such as inclusion in exocytotic vesicles (Lew and White 1987; Bowler et al. 1998; Lazarowski et al. 2000; Buckley et al. 2002).

Exposure to ATP triggers a consecutive rise in intracellular calcium concentrations and sensitizes osteoblasts to the stimulatory effects of parathyroid hormone and growth factors (Kumagai et al. 1989; Kaplan et al. 1995; Katz et al. 2006; Nishii et al. 2009). In contrast, the mechanical stress-activated calcium influx in osteoblasts is not observed after stimulation with ADP, suggesting that P2Y receptor activation is required for intracellular Ca^2+^ mobilisation (Katz et al. 2006). In osteoblasts stimulated with ATP and subsequent exposure to mechanical stress, the transient increase in calcium concentrations is followed by a fast activation of ERK1/2 and p38 MAPK pathways (Katz et al. 2006). Purinergic stimulation through P2Y_2_ receptors increases JNK1 phosphorylation, while signalling through P2X_7_ receptors results in the activation of the transcription factor FosB/AP-1 (Katz et al. 2008; Gavala et al. 2010). Activation of P2Y_1_ receptors in osteoblasts by ATP has been shown to enhance expression of receptor activator of nuclear factor-κB ligand (RANKL), resulting in enhanced osteoclast formation and resorption, while stimulation of P2Y_2_ receptors inhibits bone mineralization (Buckley et al. 2002; Gallagher and Buckley 2002).

Previously, it was reported that in a variety of different cell types including osteoblasts stimulation with high concentrations of ATP resulted in a dramatic and transient increase in membrane blebbing (Verhoef et al. 2003; Pfeiffer et al. 2004; Panupinthu et al. 2007; Roger et al. 2008; Hwang et al. 2009). The formation of membrane extensions was fully reversible and did not lead to apoptotic cell death unless receptor stimulation was prolonged (Godman et al. 1975; Panupinthu et al. 2007). Panupinthu and colleagues demonstrated that dynamic membrane blebbing in osteoblasts occurred through P2X_7_ receptors via a pathway dependent on lysophosphatidic acid and Rho-associated kinase. In this study, we asked whether purinergic stimulation of osteoblasts affects the intracellular localization of the cytoskeletal adaptor protein paxillin. We examined the subcellular localization of paxillin, since this molecule is an integral component of focal adhesions and has previously been shown to be redistributed upon mechanical loading of osteoblasts (Vatsa et al. 2008). To visualize the effects of extracellular ATP on osteoblasts, we used a green-fluorescently labelled paxillin fusion protein as a marker to assess the time course of rapid cytoskeletal rearrangements in relation to changes in calcium influx.

## Materials and methods

### Cell culture and transfection

Primary osteoblasts were isolated from bovine bones using the outgrowth method. Briefly, metacarpals obtained from steers killed at the local slaughterhouse were dissected free of muscular tissue and rinsed with phosphate-buffered saline (PBS). Periosteum pieces were removed and placed under sterile conditions in Petri dishes. Cells were cultured in BGJb medium with Fitton-Jackson modification supplemented with 10 % foetal calf serum in a humidified atmosphere of 5 % CO_2_/95 % air at 37 °C. The culture medium was replaced once a week and after 3–4 weeks a confluent and homogeneous cell culture was obtained. Osteogenic lineage was tested immunocytochemically by the expression of osteocalcin and the presence of alkaline phosphatase activity. Cells were transfected with a pEGFP-C1 expression plasmid coding for green fluorescent protein (GFP) fused to full-length human paxillin under the transcriptional control of a human cytomegalovirus promoter (a friendly gift from Dr. Irena Lavelin, Weizmann Institute, Israel). In control experiments, a pEGFP-N1 vector coding for GFP-tagged full-length human STAT1 (signal transducer and activator of transcription 1) was used. Transfection was performed with the Nanofectin kit (PAA, Pasching, Austria) according to the manufacturer’s recommendations. Twenty-four hours later, transfected cells were either left unstimulated or stimulated with a final concentration of 100 μM ATP (obtained from Carl Roth, Karlsruhe, Germany) and observed up to 60 min.

### Immunocytochemistry

The intracellular localization of endogenous paxillin in cultured osteoblasts was determined by means of indirect fluorescence microscopy. Cells were fixed with acetone/methanol (1:1) at −20 °C and permeabilized with 0.2 % Triton X-100 in PBS. After three washings in 0.1 % Triton X-100/PBS, monoclonal anti-paxillin antibody (Epitomics, Burlingame, CA, USA) was added and incubated overnight at 4 °C. The samples were washed three times in 0.1 % Triton X-100/PBS and then incubated with Texas red-labelled secondary antibody for 60 min at room temperature. Nuclei were stained with Hoechst dye. Finally, samples were rinsed three times with water and mounted in fluorescence mounting medium. The intracellular distribution of GFP-tagged paxillin was monitored by direct fluorescence microscopical examination.

### Imagine acquisition and analysis

Fluorescence images of living GFP-paxillin-expressing cells were obtained using an inverted Nikon Diaphot fluorescence microscope equipped with appropriate fluorescence filters. Fields of view for image acquisition were chosen in central regions of the plate. Images were captured with a cooled charge-coupled device (CCD) camera (Xillix, Richmond, BC, Canada) and further processed with the software program Image-Pro-Plus (Media Cybernetics, Bethesda, MD, USA). Time series captured at various time points before and after stimulation with ATP were obtained from individual cells. Fluorescence intensities over time were measured within regions of interest (ROIs) corresponding to a single paxillin-containing aggregate. For statistical analysis, all intensity time curves were individually shifted and aligned in time using the geometrical centre of the curve. The intensity signal was normalized for each ROI to the maximum intensity and averaged over all ROIs. The mean life time was determined by means of the half-width of the half amplitude method separately for the assembly and disassembly phase.

### Total internal reflection fluorescence microscopy

Osteoblasts expressing GFP-tagged paxillin were grown on glass coverslips and treated with ATP, as described. Live cell imaging using total internal reflection (TIRF) microscopy was performed in an inverted microscopy stage incubator at a temperature of 27 °C on a motorized Leica DMI 6000B microscope equipped with a TIRF illuminator module and a cooled digital 12-bit CCD camera. TIRF images were acquired every 30 s following ATP stimulation with a 100 × 1.45 Plan Neofluar oil-immersions objective at 488 nm excitation and an evanescent field with a nominal penetration depth of 100 nm. Intraobjective TIRF was obtained from 5 different cells using a 535-nm laser and a TIRF adaptor.

### Fluorescence recovery after photobleaching (FRAP)

Fluorescence recovery after photobleaching studies were conducted on living osteoblasts expressing GFP-tagged paxillin 24 h after plating on glass coverslips. An argon laser beam was focused through the fluorescence microscope (Leica TCS SP2 AOBS, Mannheim, Germany) to a Gaussian spot (λ = 488 nm) using a 100/1.3 objective. ROIs containing one or two focal adhesions were bleached by application of high-intensity laser light. The time course of the fluorescence recovery of GFP-paxillin was tracked by an attenuated monitoring beam over 200 s for each of the four cells examined. Fluorescence intensity differences between unbleached ROIs and adjoining bleached ROIs were calculated and used for an exponential curve fitting to estimate recovery times of GFP-tagged paxillin. For determination of the recovery half-life, the recovery curve normalized to the signal before bleaching was fitted to a double-exponential recovery model using the equation: $F\left( t \right) = R - A\exp \left( { - t\ln 2/t_{1/2}^{fast} } \right)-B \, \exp \left( {-t \, \ln \, 2/t_{1/2}^{slow} } \right),$ where *F*(*t*) is the fluorescence intensity at time *t*, *R* is the recovered fluorescence extrapolated to *t* → ∞, and *R* − *A* − *B* is the initial fluorescence extrapolated to *t* = 0 after photobleaching.

### Measurement of calcium concentrations

Changes in intracellular calcium concentrations in response to externally applied ATP were measured in cultured bovine osteoblasts grown on glass coverslips using Fura microfluorometry. Cells were incubated for 60 min at 37 °C with 3 μM Fura-2AM (obtained from Molecular Probes, Life Technologies, Darmstadt, Germany) in Ham’s medium supplemented with 10 mM HEPES. Loaded coverslips were placed onto the stage of an inverted Nikon Diaphot IM microscope. Intracellular Ca^2+^ signals were measured before and at various time points after exposure to 100 μM ATP using a cooled CCD imaging camera (Extended ISIS, Photonic Science, Robertsbridge, UK) controlled by a monochromator (Visitech, Sunderland, UK). A total of 90 cells from 10 independent experiments were followed up to 250 s after addition of ATP. As controls, calcium signals were measured in untreated cells (n = 12) using the same protocol. Imagines were processed using the Quanticell 700 imaging system from Applied Imaging Visitech, Sunderland, UK. The cells were sequentially illuminated with excitation wavelengths of 340 and 380 nm generated by a xenon laser. Fura-2AM fluorescence emitted at 505 nm was recorded every second with a long-pass emission filter through a Nikon 40x fluorescence objective with a numerical aperture of 0.85. For quantification, the ratio of Fura-2AM fluorescence signal intensities emitted at 505 nm with excitation at 340 nm to that with excitation at 380 nm (F_340_/F_380_) was calculated. Background values were subtracted from the mean fluorescence intensities at each wavelength before calculation of the ratio values.

### Statistical analyses

Descriptive statistics were calculated as means ± standard deviations. GFP-paxillin-containing aggregates from four representative cells were counted at different time points after addition of ATP. Differences in numbers over time were tested using one-way repeated measures analysis of variance (ANOVA) followed by a Holm-Sidak multiple pair-wise comparison test. Assembly and disassembly times for paxillin clustering were compared using the non-parametric Mann–Whitney–Wilcoxon test. Time-dependent changes in intracellular calcium levels were tested using Friedman repeated measures ANOVA on ranks followed by Tukey tests. Probability values of <5 % (*p* < 0.05) were considered statistically significant. All statistical analyses were performed with SigmaStat Version 9.0 from Systat Software.

## Results

### ATP stimulation induces paxillin-containing membrane protrusions

**Fig. 1:**
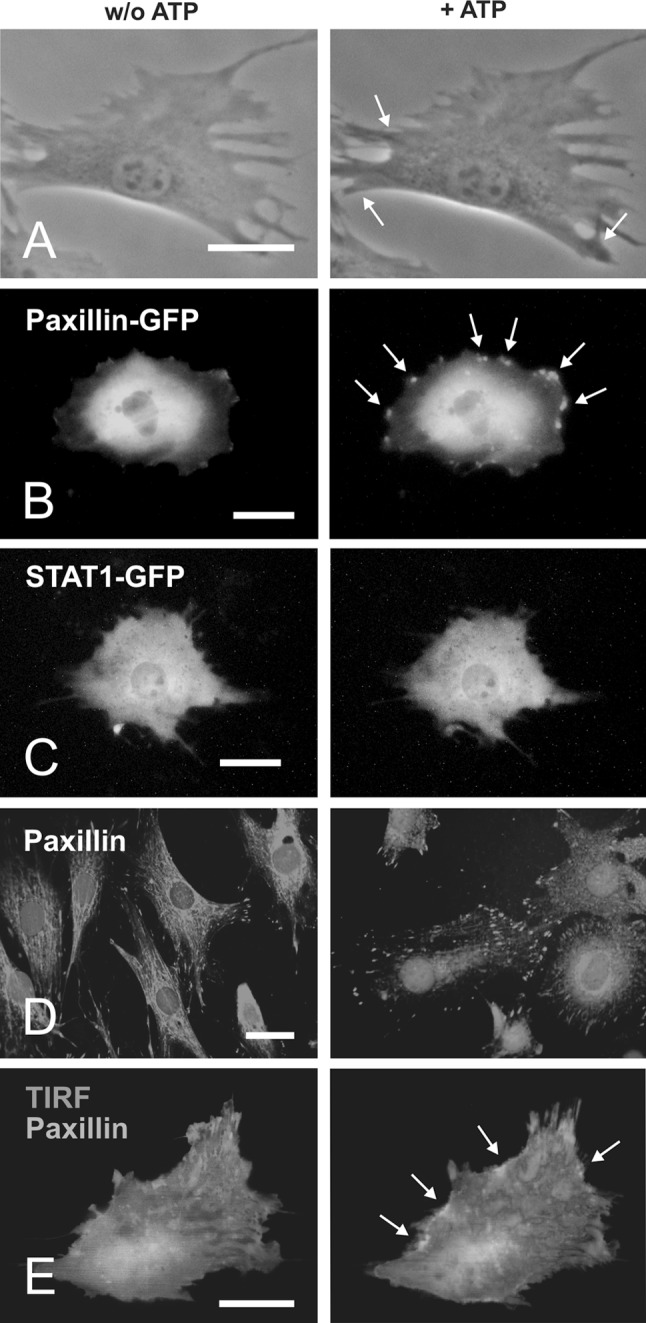
ATP stimulation induces paxillin-containing membrane protrusions on the dorsal surface of osteoblasts. **a** Phase-contrast microscopical features of a living osteoblast 0 min (*left*) and 10 min (*right*) after stimulation with 100 μM ATP. **b** Cells expressing green-fluorescent protein-tagged paxillin were stimulated with ATP (100 μM) and direct fluorescence micrographs were taken before (*left*) and 1,000 s (*right*) after exposure to ATP. Note the appearance of a bright rim of fluorescence intensity at the plasma membrane (*marked with arrows*), indicating that ATP stimulation resulted in a significant redistribution of GFP-paxillin. **c** Control experiments using GFP-fusions of STAT1 transcription factor instead of paxillin revealed no such GFP redistribution of following ATP exposure. **d** Localization of endogenous paxillin in focal adhesions as visualized by indirect fluorescence microscopy. Cells either left untreated (*left*) or treated with ATP (*right*) were fixed with formalin and permeablized with 0.2 % Triton X-100/PBS. Nuclei were stained with Hoechst dye before cells were subsequently incubated with anti-paxillin antibodies followed by Texas red-labelled secondary antibodies. Note the localization of paxillin in focal adhesions in permeabilized cells. **e** Membrane protrusions induced by ATP stimulation reside predominantly at the dorsal and not the ventral surface of the cells. To assess the appearance of paxillin at the ventral cell site, total internal reflection (TIRF) microscopy was used in a GFP-paxillin-expressing osteoblast before (*left*) and 10 min after addition of ATP to the culture medium (*right*). However, there were no corresponding TIRF signals (*marked in red*) despite a significant redistribution of paxillin (epifluorescence in *green*) at the cell periphery (*marked with arrows*), thus excluding the possibility that the newly formed membrane protrusions were exclusively located at the ventral cell surface (*bar* in each micrograph 10 μm). (Color figure online)

To determine whether stimulation with high concentrations of ATP affects the organization of the cytoskeleton in bovine osteoblasts, we first performed phase-contrast microscopy and detected the appearance of lamellipodial extensions at the cytoplasmic rim of the cell (Fig. 1a). Next, we determined the intracellular localization of the focal adhesion protein paxillin in living osteoblasts by means of fluorescence microscopical analysis (Fig. 1b). In transfected cells expressing green-fluorescent protein-tagged paxillin the bulk fluorescence activity was detected in a homogeneous pattern throughout the cytoplasm, while the nuclei were typically excluded (Fig. 1b). Only a minor fraction of the recombinant fusion protein was localized in focal adhesions at the cell periphery. After stimulating cells with a final concentration of 100 μM ATP, we observed the formation of numerous membrane protrusions preferentially at the cell periphery. Using direct fluorescence microscopy these structures showed a bright staining of fluorescently labelled paxillin. Typically, the GFP-fusion protein accumulated in either lamellipodial protrusions or multiple small aggregates at the rim of the cell (Fig. 1b). No such GFP-containing focal membrane structures were visualized in ATP-stimulated osteoblasts expressing STAT1-GFP, which were used as controls (Fig. 1c)

.

Immunofluorescence staining of endogenous paxillin in permeabilized, formaldehyde-fixed cells before and after ATP stimulation demonstrated the association of paxillin with focal adhesions, where it localized at the tips of stress fibres (Fig. 1d). Closer inspection at higher magnification revealed that frequently filamentous structures terminated in paxillin-containing focal adhesions, while occasionally some focal adhesions seemed to lack obvious continuity to the filamentous network. Due to permeabilization of cells there was no homogeneous paxillin immunostaining throughout the cytoplasm, as opposed to the diffuse cytoplasmic localization of GFP-paxillin in living, non-permeabilized cells. Using the same staining protocol as for untreated cells we failed to detect a subsequent redistribution of native paxillin molecules into membrane protrusions in ATP-stimulated osteoblasts, probably because these structures did not resist fixation and cell permeabilization.

To determine whether or not paxillin-containing membrane structures localize predominantly to the ventral surface of the cell, where they may play a role in the anchorage to the substratum, we performed total internal reflection (TIRF) microscopy. TIRF technology uses evanescent waves that selectively illuminate and excite GFP fluorophores in restricted regions of the specimen adjacent to the glass-water interface. This evanescent field penetrates only about 100 nm into the cell, as it decays exponentially away from the source (Berginski et al. 2011). For TIRF, we treated the GFP-expressing osteoblasts with ATP as described. Although membrane protrusions were seen in GFP epifluorescence induced by ATP stimulation, we rarely detected co-localization with TIRF signals and thus excluded the possibility that the GFP-paxillin aggregates were preferentially located at the ventral surface of the cell (Fig. 1e).

### Dynamics of paxillin clustering in ATP-induced membrane protrusions

**Fig. 2:**
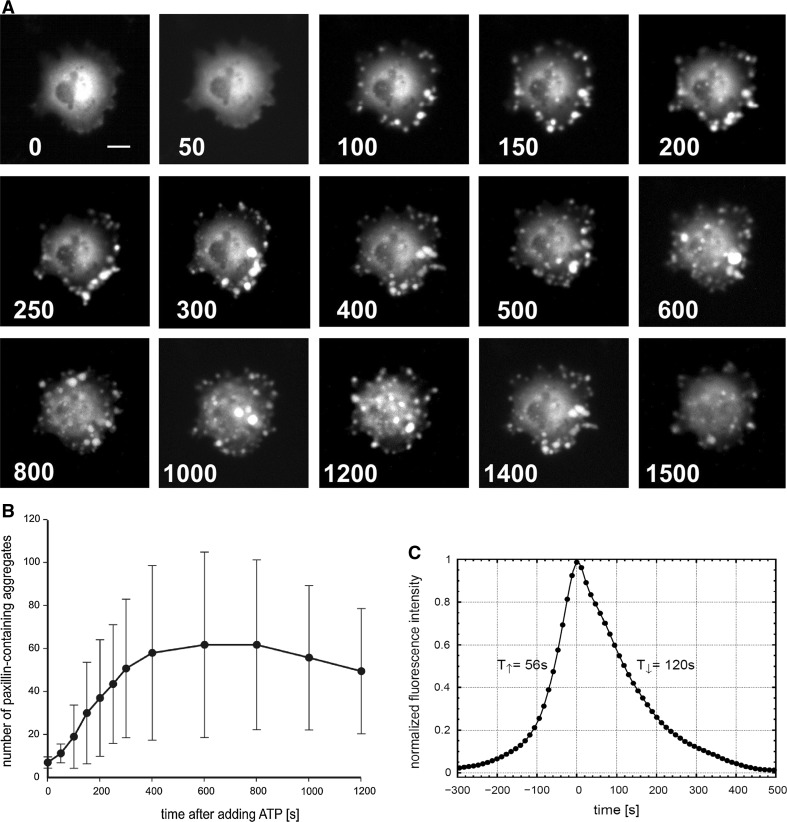
Assembly of paxillin-containing focal aggregates in ATP-stimulated, living osteoblasts. **a** Time-lapse video-microscopy was performed to demonstrate the time course of ATP-induced recruitment of GFP-paxillin to membrane protrusions. Cells expressing GFP-paxillin (n = 25) were stimulated 24 h post-transfectionem with 100 μM ATP and the intracellular localization of GFP-paxillin was monitored over time for the indicated time points, given in seconds, after the stimulation with the ATP. Shown is the formation of multiple paxillin-containing aggregates in a representative cell, which are scattered throughout the dorsal cell surface over at least 1,200 s after addition of ATP (*bar* 10 μm). **b** Time-dependent changes in the number of paxillin-containing aggregates in ATP-treated osteoblasts as determined from 4 independent experiments. **c** Averaged kinetics of paxillin redistribution upon stimulation of cells with ATP demonstrated the rapid formation and slightly slower disruption of reversible GFP-paxillin-containing aggregates. Changes in fluorescence intensity over time were measured in a single membrane protrusion using direct fluorescence microscopy and data were normalized. The life time of individual paxillin-containing aggregates was determined by means of the half-width of the half amplitude method separately for the assembly and disassembly phase

To confirm the intracellular redistribution of paxillin observed in ATP-stimulated cells, we performed time-lapse imaging analysis of osteoblasts expressing recombinant paxillin fused to GFP. Cells were exposed to 100 μM ATP and the dynamic redistribution of the fluorescently labelled protein was monitored over time by means of direct fluorescence microscopy (Fig. 2a). The imagine sequences demonstrated that within 50 s after adding ATP there was no gross change in the nearly pancellular, homogeneous localization of GFP-tagged paxillin. However, 100 s after addition the nucleotide we observed the de novo formation of fluorescently labelled membrane protrusions, which appeared preferentially at the periphery of the cells. The number and size of the GFP-paxillin-containing focal aggregates further increased up to 600 s after the agonist had been added (Fig. 2a, b). The total amount of GFP-tagged paxillin incorporated into the aggregates then slowly decreased. This time-dependent change in the number of clustered paxillin-containing foci was statistically significant (*p* < 0.001).

However, we noticed that the half-life of an individual labelled fluorescence dot was obviously much shorter. Most of the existing paxillin aggregates faded away, while only a minority converged to confluent lamellipodial extensions. Taken together, time-lapse imaging of GFP-tagged paxillin in transiently transfected osteoblasts confirmed our first observation that upon stimulation of cells with ATP, the cytoskeletal protein paxillin is redistributed into newly formed membrane protrusions. Moreover, fluorescence microscopy of living cells revealed the highly dynamic nature of paxillin recruitment into these focal membrane structures, which appeared as flashing dots at the dorsal surface of cells.

### Assembly/disassembly kinetics of paxillin-containing focal membrane structures

Given the ephemeral association of paxillin into focal clusters at the cell periphery resulting from stimulation with ATP, we next aimed to determine the exact recruitment kinetics of paxillin to these sites. Osteoblasts transiently expressing GFP-paxillin were stimulated with 100 μM ATP and subsequently tracked for incorporation of GFP epifluorescence by means of time-lapse microscopy (Fig. 2b). We found that in ATP-stimulated cells the build-up as well as the subsequent break-down of GFP-paxillin-containing aggregates was a very rapid process as compared to the stability of focal adhesions in long-cultured osteoblasts. Typically, the assembly phase for paxillin incorporation was shorter than its disassembly as measured in four independent experiments (49 ± 9 s vs. 101 ± 28 s, *p* = 0.029), suggesting that the exchange dynamics of paxillin is highly regulated.

### Exchange rates of paxillin at mature focal adhesions

**Fig. 3:**
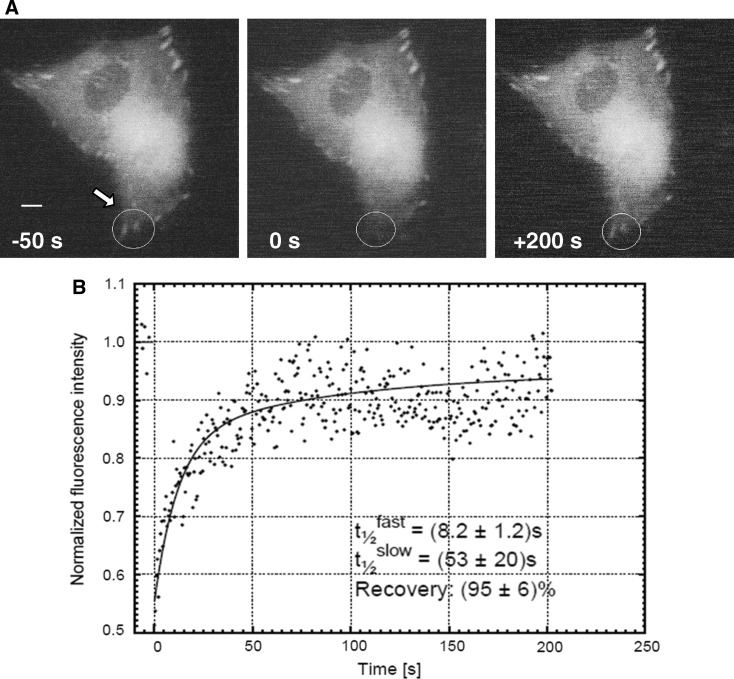
Rapid exchange of paxillin in mature focal adhesions in cultured bovine osteoblasts. **a** Fluorescence recovery after photobleaching (FRAP) in a typical GFP-paxillin-expressing cell demonstrated the rapid incorporation of paxillin in stable focal adhesions. The bleached region is marked with an *arrow*. Shown is a cell 50 s before (−50 s) as well as immediately (0 s) and 200 s (+200 s) after photobleaching (*bar* 5 μm). **b** FRAP data of GFP-paxilllin-expressing cells were best fitted with a double-exponential function with two different kinetic constants, indicating the presence of a fast and slow exchange process. Depicted are the recovery half-lifes for the two exchange rates. Total fluorescence recovery was 95 ± 6 %, suggesting that there is no significant immobile fraction of paxillin

Adaptor molecules such as paxillin are soluble cytosolic components of the cytoskeleton which participate in dynamic exchange processes between a membrane-associated and an unbound state (Wolfenson et al. 2011). In order to assess the turn-over of paxillin at mature focal adhesion plaques, we used fluorescence recovery after photobleaching (FRAP) technology in living bovine osteoblasts expressing GFP-paxillin. Single focal adhesions localized at the periphery of the cell were bleached by high-intensity laser irradiation in a Gaussian spot and the recovery of GFP epifluorescence in a region of interest (ROI) was monitored over a period of 200 s (Fig. 3a). The time course of the fluorescence recovery in the ROI was tracked by attenuated monitoring beam and the resulting curves were fitted by non-linear regression analysis. The FRAP data were best fitted when fluorescence recovery was composed of two independent mechanisms: one being the recovery by relatively fast lateral diffusion and the other by slow exchange (Fig. 3b). The mean recovery half-life of GFP-paxillin in the early phase taken from four independent measurements was 8.2 s with an uncertainty of 1.2 s, while corresponding values in the later phase were 53 ± 20 s. These data compared well with the estimated formation time of focal adhesions in ATP-stimulated osteoblasts, as described above. The recovery of GFP fluorescence in the ROI reached 95 ± 6 % of its initial intensity, suggesting that there is no significant immobile fraction of paxillin. In conclusion, the accumulation of GFP-paxillin in newly formed membrane clusters and its incorporation in pre-existing mature plaques are both rapid processes that occur within 1 min.

### Time course of calcium response in ATP-stimulated osteoblasts

**Fig. 4:**
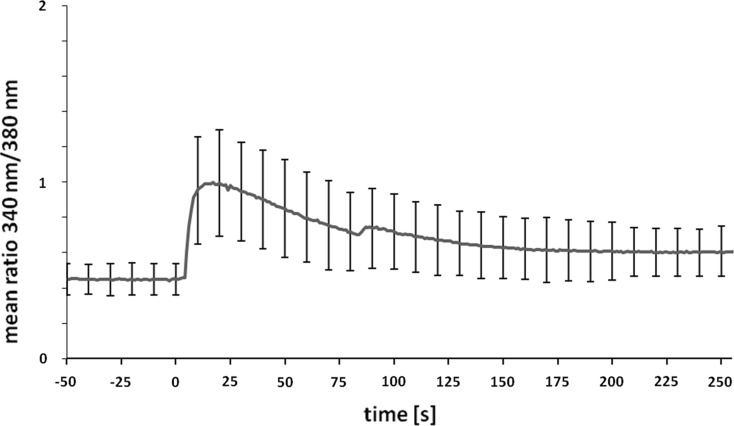
Time course of calcium influx in osteoblasts exposed to high concentrations of ATP. Cultured cells (n = 90) were loaded with Fura-2AM and subsequently stimulated with 100 μM of the nucleotide (defined as time point t = 0). Fura-2AM fluorescence signal intensities emitted at 505 nm were measured using excitation wavelengths of 340 and 380 nm, respectively. The means and standard deviations of the ratio F_340_/F_380_ were calculated and plotted over time before and after stimulation with ATP

The next step was to determine the time course of calcium transients in response to ATP stimulation in isolated bovine osteoblasts using Fura-2AM imaging technique. Intracellular calcium concentration profiles were measured over a 50-second baseline period followed by 200 s of stimulation with either 0 μM or 100 μM ATP (Fig. 4). Although there was some heterogeneity in the cytosolic [Ca^2+^]_i_ profiles of individual cells even within the same culture dish, it was clearly found that osteoblasts showed significant calcium increments during the stimulation phase as compared to pre-stimulation baseline (*p* < 0.001). Serial images demonstrated that peak values were typically reached within 20 s after adding the purinergic agonist to the cells. The fast ATP-induced calcium rise was followed by a slow decrement, which lasted approximately 150 s. Then the Fura-2AM fluorescence signal intensity approximated a steady level, which however was higher than before stimulation. A similar increment in intracellular calcium concentration was observed, when cells were stimulated with bradykinin, which is known to cause calcium inflow from the extracellular milieu (data not shown). As expected, no calcium response was detected in cells exposed to Ham’s solution alone (data not shown).

## Discussion

In the present study we report on the de novo accumulation of the multi-domain scaffold protein paxillin in membrane protrusions resulting from stimulation of bovine osteoblasts with the purinergic agonist ATP. In addition, we assessed the rapid incorporation and dynamic turn-over of GFP-tagged paxillin in mature focal adhesions and determined the time course of intracellular calcium concentrations following exposure of cells to ATP. The data obtained allowed us to determine the occurrence of calcium transients in relation to cytoskeletal rearrangements in ATP-treated bone-derived cells.

Adenosine 5`-triphosphate, an important regulatory molecule in bone tissue homeostasis, is known to induce formation of inositol 3-phosphate and elevation of intracellular calcium levels through acting on either G-protein-coupled P2Y receptors or ligand-gated ion channels (Gallagher and Buckley 2002; Orriss et al. 2006). The pivotal role of extracellular ATP in the regulation of skeletal homeostasis is well established, but less is known about the molecular mechanisms behind the diverse functions of ATP in bone-derived cells. Particularly, the morphological changes and cytoskeletal reorganization following exposure to high concentrations of this nucleotide are not well understood (Panupinthu et al. 2007).

The major finding of this study is the rapid assembly of paxillin-containing focal aggregates in osteoblasts following stimulation with this nucleotide. By video tracking of bone-derived cells expressing a GFP-fusion protein of paxillin, we observed a fast and transient accumulation of GFP-tagged paxillin in lamellipodial extensions and numerous newly formed membrane excrescences, which appeared as distinct bright spots at the periphery or the dorsal surface of the cells when visualized by direct fluorescence microscopy. Interestingly, the ATP-induced, paxillin-containing focal aggregates were rather ephemeral structures predominately at the dorsal cell surface, which emerged already within 100 s after addition of the nucleotide. The mean life time of a single membrane spot was determined to be <4 min, including both rapid assembly and disassembly. In general, the build-up of an individual aggregate was significantly shorter than its subsequent break-down, with the latter lasting typically twice as long as the preceding accumulation phase. However, beside the fluorescently labelled membrane clusters, there was still a diffuse background staining throughout the cytoplasm, indicating that the majority of paxillin molecules remained in an unbound cytosolic state, while only a minor fraction of the total cellular paxillin was incorporated into the newly formed fluorescence spots.

Although the accumulation of GFP-paxillin in membrane sub-domains was a fast process occurring within 20 min of ATP stimulation, the turn-over of paxillin at a single mature focal adhesion plaque was even much faster. By analyzing our FRAP data from GFP-paxillin-expressing, unstimulated osteoblasts, the recovery of fluorescence intensity at mature focal adhesions was best fitted with a biphasic fluorescence curve, which probably resulted from the superposition of a fast cytoplasmic diffusion-controlled process and a comparably slower exchange rate of loosely bound paxillin that was reversibly incorporated in focal adhesions.

To determine the time course of calcium changes in relation to the observed cytoskeletal reorganization, we performed calcium measurements after treating cells with ATP. Binding of extracellular ATP to P2 receptors is known to increase intracellular calcium levels (Kumagai et al. 1989; Kaplan et al. 1995; Katz et al. 2006; Nishii et al. 2009), and our study confirms that in osteoblasts treated with ATP a transient rise in intracellular calcium levels occurs, ultimately leading to the recruitment of paxillin to newly formed membrane protrusions. Clearly, the increase in calcium concentrations preceded the incorporation of paxillin in newly formed focal membrane clusters. While [Ca^2+^]_i_ concentrations peaked <25 s after adding the nucleotide, the initial recruitment of paxillin to membrane aggregates did not commence within the first 50 s. Thus, the rapid and transient rise in cytoplasmic [Ca^2+^]_i_ is an early process in ATP signalling, which is nearly accomplished before the dramatic changes in paxillin redistribution occur. At later stages following ATP stimulation, the clustering of the focal adhesion component paxillin gradually decreased and ultimately GFP-paxillin was no longer retained in individual foci on the dorsal surface of the cells. Thus, the recruitment of paxillin to restricted membrane sub-domains upon ATP stimulation is by one or two orders of magnitude slower than its rapid turn-over in mature focal adhesions. Nevertheless, the exchange of paxillin molecules at stable focal adhesions and their dynamic redistribution to nascent membrane protuberances induced by ATP stimulation are both fast processes, which are supposed to change the morphology of the cell.

Our presented FRAP data confirm previous studies in other cell lines, showing that paxillin exists in different subcellular fractions such as cytoplasmic, focal-adhesion-associated and juxtamembrane pools that interchange rapidly between each other (Vatsa et al. 2008; Wolfenson et al. 2009). Paxillin is continuously recruited from a highly mobile cytoplasmic fraction and, conversely, released from the focal adhesion-associated scaffold, thereby producing a concentration gradient from the focal adhesion area to the surrounding juxtamembrane cytoplasm (Wolfenson et al. 2009). The clustering of paxillin at the cell periphery and its rapid exchange with a mobile pool in the cytoplasm probably allows for a flexible response towards structural changes engaged in ATP-mediated cell motility and bone remodelling.

Recently, it was shown by Wolfenson and colleagues that actomyosin-generated tension regulates the molecular kinetics of focal adhesion (Wolfenson, et al. 2011). The authors have demonstrated that inhibition of myosin II contractility facilitates the release of vinculin from focal adhesions, while dissociation of paxillin and zyxin is attenuated. The dynamics of paxillin in mature focal adhesions appear to be independent from its association with vinculin, suggesting that the interaction between paxillin and vinculin is differentially regulated in nascent and mature focal adhesions (Humphries et al. 2007). Beside its structural role in the formation of nascent focal adhesions, paxillin may also be involved in the efficient disassembly of focal adhesion complexes, as indicated by its transient residency time in mature focal adhesions and its role in destabilizing adhesions in cells that lack expression of functional paxillin (Webb et al. 2004).

Our observation linking paxillin to the rapid formation and disassembly of membrane protuberances illustrates the central role of this scaffold protein in the cytoskeletal organization of osteoblasts following treatment with ATP. Other cytoskeletal proteins such as vinculin and calmodulin have been shown to redistribute in a similar manner (Roger et al. 2008; Tan et al. 2010). In HEK cells expressing rat P2X_7_ receptors, Roger and co-workers have reported that calmodulin binds constitutively to closed P2X_7_ receptors and dynamically during channel activation, thereby enhancing the inflow of calcium ions. Moreover, the authors identified a Ca^2+^-dependent calmodulin binding motif in the carboxy-terminus of P2X_7_ receptor, which upon mutation resulted in delayed membrane blebbing. In fibroblasts, expression of the constitutively active Ca^2+^/calmodulin-dependent protein kinase CaMK-II has been shown to inhibit paxillin incorporation into focal adhesions, reduce cell attachment and induce tyrosine dephosphorylation of focal adhesion kinase (FAK) and paxillin (Easley et al. 2008). In corneal epithelial cells exposed to ATP and other nucleotides a rapid increase in the phospho-protein levels of paxillin at the two phosphorylation sites Y31 and Y118 was observed (Klepeis et al. 2004).

Taken together, our data show that stimulation of osteoblasts with extracellular ATP induces the formation of short-living, paxillin-containing membrane protuberances. The reversible paxillin clustering both in ATP-induced membrane structures and mature focal adhesions indicates that paxillin is engaged in heterogeneous signalling complexes that differ from each other with respect to subcellular localization and possibly molecular architecture. The fugacious nature of the ligand-mediated membrane blebbing and the dynamic exchange of paxillin both reflect the high level of complexity in the spatiotemporal organization of cytoskeletal proteins at the plasma membrane, suggesting that paxillin-containing clusters display ‘hot spots’ of cell–matrix interactions and signal transduction. Since we do not know the exact function and regulation of paxillin in ATP-stimulated osteoblasts, our finding of the high mobility of this adaptor molecule emphasizes the need for further, more detailed investigations.
